# Effect of Complete Revascularization in STEMI: Ischemia-Driven Rehospitalization and Cardiovascular Mortality

**DOI:** 10.3390/jcm14134793

**Published:** 2025-07-07

**Authors:** Miha Sustersic, Matjaz Bunc

**Affiliations:** 1 Department of Cardiology, University Medical Centre Ljubljana, Zaloska 7, 1000 Ljubljana, Slovenia; matjaz.bunc@kclj.si; 2 Institute of Pathological Physiology, Medical Faculty, University of Ljubljana, Vrazov trg 2, 1000 Ljubljana, Slovenia

**Keywords:** ST-elevation myocardial infarction, multivessel coronary artery disease, complete percutaneous revascularization, ischemia-driven rehospitalization, cardiovascular mortality

## Abstract

**Background**: Patients with ST-elevation myocardial infarction (STEMI) and multivessel coronary artery disease (MVD) who undergo complete revascularization (CR) have a more favorable prognosis than those who receive incomplete revascularization (IR), as evidenced by recent randomized controlled trials. Despite the absence of a survival benefit associated with CR in these trials, positive outcomes were ascribed to combined endpoints, such as repeat revascularization, myocardial infarction, or ischemia-driven rehospitalization. In light of the significant burden that rehospitalization from STEMI imposes on healthcare systems, we examined the long-term effects of CR on ischemia-driven rehospitalization and cardiovascular (CV) mortality in STEMI patients with MVD. **Methods**: In our retrospective study, we included patients with STEMI and MVD who underwent successful primary percutaneous coronary intervention (PCI) at the University Medical Centre Ljubljana between 1 January 2009, and 11 April 2011. The combined endpoint was ischemia-driven rehospitalization and CV mortality, with a minimum follow-up period of six years. **Results**: We included 235 participants who underwent CR (N = 70) or IR (N = 165) at index hospitalization, with a median follow-up time of 7 years (interquartile range 6.0–8.2). The primary endpoint was significantly higher in the IR group than in the CR group (47.3% vs. 32.9%, log-rank *p* = 0.025), driven by CV mortality (23.6% vs. 12.9%, log-rank *p* = 0.047), as there was no difference in ischemia-driven rehospitalization rate (log-rank *p* = 0.206). Ischemia-driven rehospitalization did not influence CV mortality in the CR group (*p* = 0.49), while it significantly impacted CV mortality in the IR group (*p* = 0.03). After adjusting for confounders, there were no differences in CV mortality between CR and IR groups (*p* = 0.622). Predictors of the combined endpoint included age (*p* = 0.014), diabetes (*p* = 0.006), chronic kidney disease (CKD) (*p* = 0.001), cardiogenic shock at presentation (*p* = 0.003), chronic total occlusion (CTO) (*p* = 0.046), and ischemia-driven rehospitalization (*p* = 0.0001). Significant risk factors for the combined endpoint were cardiogenic shock at presentation (*p* < 0.001), stage 4 kidney failure (*p* = 0.001), age over 70 years (*p* = 0.004), female gender (*p* = 0.008), and residual SYNTAX I score > 5.5 (*p* = 0.017). **Conclusions**: Patients with STEMI and MVD who underwent CR had a lower combined endpoint of ischemia-driven rehospitalizations and CV mortality than IR patients, but after adjustments for confounders, the true determinants of the combined endpoint and risk factors for the combined endpoint were independent of the revascularization method.

## 1. Introduction

ST-segment elevation myocardial infarction (STEMI) patients with multivessel coronary artery disease (MVD) have a worse prognosis than STEMI patients with culprit-only involvement [1]. In the past decade, there has been a debate regarding the efficacy of performing complete revascularization (CR) on STEMI patients with MVD during the index hospitalization/procedure or whether non-culprit lesions should be treated solely based on symptoms or proven significant myocardial ischemia in the delayed percutaneous coronary procedure (PCI) [2,3,4,5]. Although prospective randomized studies have demonstrated the advantages of CR in terms of their outcomes, none have demonstrated a benefit in terms of hard outcomes, such as mortality, to date [6,7,8,9,10]. It is acknowledged that certain noninvasive tests, such as stress tests without imaging, may not be diagnostic for certain patients. However, other methods with greater sensitivity and specificity are more expensive, more invasive, and require radiation or contrast media, as well as being more difficult to access [11]. There is also a lack of certainty about the appropriate time to consider patients who have a significant non-culprit disease as patients with chronic stable angina after STEMI. Patients with chronic ischemic heart disease do not all benefit from percutaneous revascularization methods, as previously demonstrated, and optimal medical therapy may be equivalent to invasive coronary treatment [12,13,14]. The treatment of patients may be further enhanced by the detection of unstable plaques using intravascular plaque imaging [15,16,17,18]. In this study, we investigated the long-term effects of CR during the index hospitalization of STEMI and MVD patients on ischemia-driven rehospitalization and CV mortality. Due to the scarcity of long-term follow-up data on patients with STEMI and MVD, this was deemed necessary. In this data set, we have already demonstrated that the method of revascularization did not have a substantial impact on all-cause or cardiovascular mortality [19].

## 2. Materials and Methods

### 2.1. Study Design and Participants

We retrospectively included a group of consecutive STEMI patients with MVD who had primary PCI at the University Medical Center Ljubljana (UMCL), Slovenia. These patients had a STEMI diagnosis (the diagnosis was made according to the fourth universal definition of myocardial infarction) [20]. All coronary angiographies were reviewed by two experienced interventional cardiologists for successful PCI of the culprit coronary artery—TIMI flow ≥ 2 and at least one non-culprit coronary artery stenosis ≥ 50% with a diameter > 2 mm. The revascularization during index hospitalization had to be with PCI only. Patients who underwent PCI during their index hospitalization and had all significant stenoses revascularized were placed in the complete revascularization (CR) group; those who did not were placed in the incomplete revascularization (IR) group. We determined the residual SYNTAX I score (Synergy Between PCI With Taxus and Cardiac Surgery score I) for each patient following PCI of the culprit coronary artery in order to assess the complexity of non-culprit coronary artery disease. There were no other exclusion criteria to represent an everyday clinical practice. We noted CV risk factors and comorbidities. Every aspect of the PCI procedure was recorded, including the laboratory values upon admission and the most significant deviations during the hospital stay, the medical treatment both at admission and after discharge, and the requirement for a transfusion. Cardiogenic shock was characterized by persistently low blood pressure, end-organ hypoperfusion, or the requirement for an intra-aortic balloon pump. We planned a follow-up period of at least 6 years for all patients. The previous publication provided details regarding data collection [19]. The study’s primary endpoints were ischemia-driven rehospitalization and cardiovascular mortality. Any rehospitalization (planned or unplanned) to rule in or rule out ischemic heart disease, during which further invasive or noninvasive tests were performed for the condition, was referred to as ischemia-driven rehospitalization. Planned ischemia-driven rehospitalization was defined as rehospitalization due to significant ischemia found on a noninvasive test after index hospitalization. Unplanned rehospitalization was defined as rehospitalization due to symptoms of myocardial ischemia, during which noninvasive or invasive tests to reveal myocardial ischemia were executed after index hospitalization. Cardiovascular death was defined as fatal acute myocardial infarction, sudden cardiac death, death due to heart failure, stroke, cardiovascular procedure, cardiovascular hemorrhage, or other cardiovascular causes. We conducted the study in accordance with the Declaration of Helsinki and received approval from the Slovene Medical Ethics Committee. Since we obtained the data retrospectively and maintained patient confidentiality, we did not require written informed consent.

### 2.2. Statistical Analysis

We reported descriptive statistics as mean, standard deviation (SD), and counts (percentages). We tested the comparison between groups using the Mann–Whitney and Fisher exact tests. We used the Kaplan–Meier method for non-parametric survival analysis. We used the log-rank test to compare the survival distributions of two or more independent groups. Using a Cox proportional hazards model, we evaluated the effect of several factors on the clinical endpoint. Factors included in the multivariable Cox model were selected based on established clinical relevance, the prior literature, and potential association with cardiovascular mortality or rehospitalization (age, gender, smoking history, diabetes, dyslipidemia, chronic kidney disease, and residual SYNTAX I score), including those used as exclusion criteria in other major STEMI and MVD trials (prior myocardial infarction, PCI, coronary artery bypass graft surgery (CABG), known CTO before inclusion, and cardiogenic shock at presentation). To determine the optimal age, chronic kidney disease, and residual SYNTAX I score cutoffs for risk stratification, we performed exploratory data mining using a maximally selected rank statistic and outcome-based thresholding. In order to demonstrate the impact of each component on the combined endpoint occurrence, we computed hazard ratios for each individual factor. We tested all hypotheses at the pre-specified significance level of less than 0.05. We performed data analysis and processed statistics using the software package R (R version 4.0, R Foundation for Statistical Computing, Vienna, Austria).

## 3. Results

### 3.1. Baseline Characteristics

**Scheme 1:**
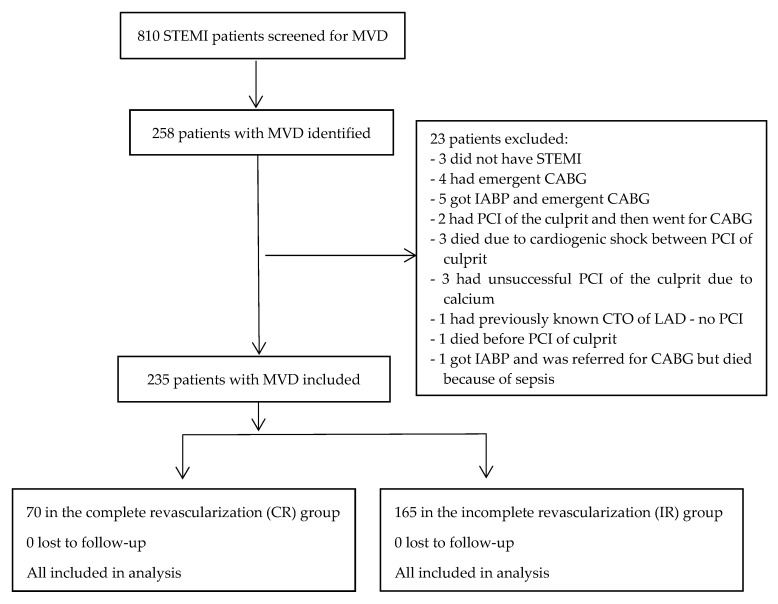
Patient flow chart [19].

Between 1st January 2009 and 3rd April 2011, we identified 810 STEMI patients. A total of 258 (31.9%) patients had MVD, but 23 (9%) patients were excluded, so the study population comprised 235 (91%) individuals. Of these, 70 (30%) underwent CR, while 165 (70%) underwent IR (patient flow chart, Scheme 1). The two groups had similar demographic data, comorbidities, chronic therapies prior to inclusion in the study and at discharge, laboratory values, culprit coronary artery involvement, and LVEF after index PCI (Table 1 and Supplementary Table S1). There was a higher burden of non-culprit coronary artery stenosis (*p* = 0.015), CTO (*p* < 0.001), and residual SYNTAX I score in the IR group (*p* = 0.011), while the CR group had more coronary interventions (*p* < 0.001) and the severity of non-culprit stenosis was higher (*p* = 0.015) (Table 1 and Supplementary Table S4). The data were previously reported [19].

### 3.2. Ischemia-Driven Rehospitalization and Cardiovascular Mortality

**Figure 1:**
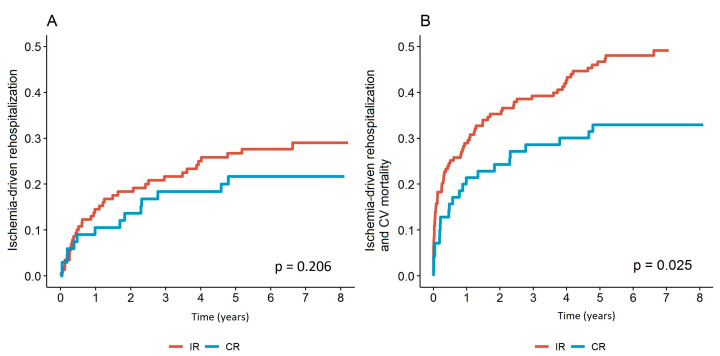
Rehospitalization rate and primary combined endpoint occurrence of ischemia-driven rehospitalization and CV mortality. (**A**) Ischemia-driven rehospitalization in the CR and IR groups; (**B**) ischemia-driven rehospitalization and CV mortality in the CR and IR groups. Legend: CR: complete revascularization; IR: incomplete revascularization.

**Figure 2:**
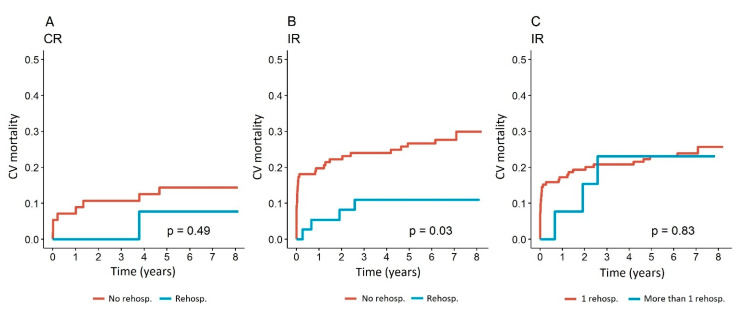
Effect of ischemia-driven rehospitalizations on CV mortality. (**A**) CV mortality of the CR group: comparison between subjects without ischemia-driven rehospitalization and subjects with ischemia-driven rehospitalization; (**B**) CV mortality of the IR group: comparison between subjects without ischemia-driven rehospitalization and subjects with ischemia-driven rehospitalization; (**C**) CV mortality of the IR group: comparison between subjects who had only one and subjects who needed two or more ischemia-driven rehospitalizations. Legend: CR: complete revascularization; IR: incomplete revascularization.

There were no ischemia-driven rehospitalization plans for further myocardial revascularization in the release documents in the IR patient group at index hospitalization. The likelihood of ischemia-driven rehospitalization was comparable between the CR and IR groups (*p* = 0.206) (Figure 1A). There were also no significant differences in the incidence of different forms of myocardial ischemia (stable angina pectoris, acute coronary syndrome, heart failure due to ischemic heart disease) or other causes for admission to exclude significant myocardial ischemia (*p* = 0.519). In the case of rehospitalization, the method of further revascularization of the myocardium was not different between the CR and IR groups (Table 2). While hospitalization had a substantial influence on cardiovascular mortality in the IR group (*p* = 0.03) (Figure 2B), rehospitalization had no effect on cardiovascular mortality in the CR group (*p* = 0.49) (Figure 2A). After the first rehospitalization, further hospital treatments in the IR group did not have a significant impact on cardiovascular mortality (*p* = 0.83) (Figure 2C). The incidence of ischemia-driven rehospitalization and cardiovascular mortality in the CR group was 23 of 70 (32.9%; 95% CI for the average follow-up time to the event 5.5–6.3 years) and 78 of 165 in the IR group (47.3%; 95% CI for the average follow-up time to the event 4.5–5.1 years). The survival curves for the primary endpoint of the CR and IR groups significantly differed at the end of the follow-up (log-rank *p* = 0.025) (Figure 1B).

**Figure 3:**
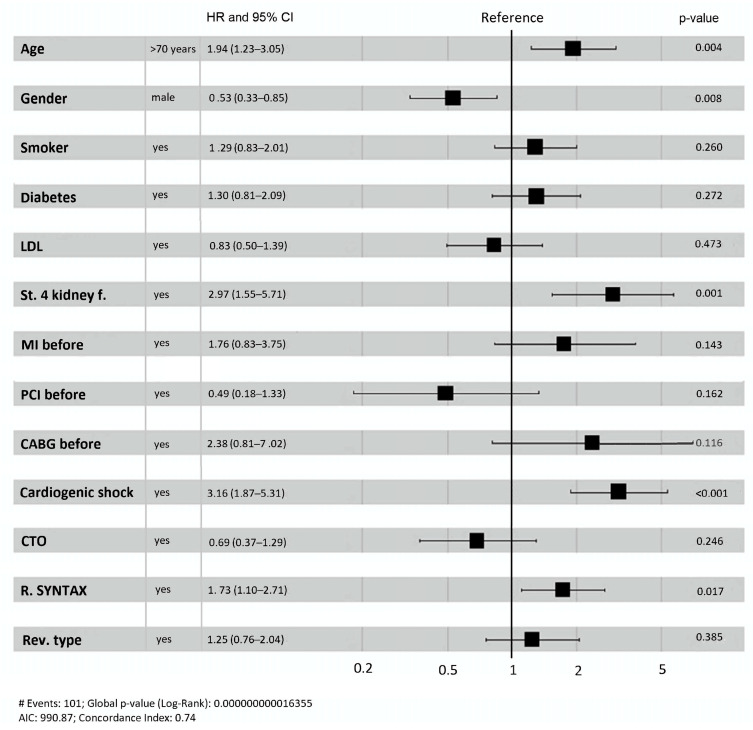
Risk factors for ischemia-driven rehospitalization and CV mortality. LDL—low-density lipoprotein, MI before—myocardial infarction before index hospitalization, PCI before—percutaneous coronary intervention before index hospitalization, CABG before—coronary artery bypass graft surgery before index hospitalization, CTO—chronic total occlusion, R. SYNTAX—residual SYNTAX I score, IR—incomplete revascularization, St. 4 kidney f.—stage 4 or 5 kidney failure, HR—hazard ratio, CI—confidence interval.

CR did not demonstrate any improvements in ischemia-driven rehospitalization and cardiovascular mortality after accounting for confounders (Table 3). The risk of combined endpoint occurrences was observed to increase with age (*p* = 0.014), diabetes (*p* = 0.006), chronic kidney disease (*p* = 0.001), cardiogenic shock at presentation (*p* = 0.003), chronic total occlusion (*p* = 0.046), and the necessity for ischemia-driven rehospitalization (*p* < 0.0001) (Table 3). In addition, we endeavored to ascertain which entity increases the likelihood of the primary combined endpoint of ischemia-driven rehospitalization and CV mortality in patients with STEMI and MVD. IR does not significantly increase the risk of the primary combined endpoint (HR 1.25, *p* = 0.385). However, it was significantly increased by the following factors: a residual SYNTAX I score greater than 5.5 (HR 1.73, *p* = 0.017), female gender (HR 1.88, *p* = 0.008), age of more than 70 years (HR 1.94, *p* = 0.004), stage 4 or 5 chronic kidney disease (HR 2.97, *p* = 0.001), and cardiogenic shock at the inclusion (HR 3.16, *p* < 0.001) (Figure 3). Risk analysis by residual SYNTAX I score categories for the composite endpoint of ischemia-driven rehospitalization and CV mortality supported the cutoff value of 5.5 for residual SYNTAX I score (Supplementary Figure S1 and Supplementary Table S5).

### 3.3. Complications

The incidence of serious complications due to PCI during index hospitalization was 4.3% in the CR group and 9.7% in the IR group. In the CR group, all complications occurred during PCI of the culprit coronary artery, whereas in the IR group, only one subject experienced a complication during PCI of a non-culprit coronary artery. There were no significant differences in the incidence of complications between the CR and IR groups during index hospitalization (*p* = 0.199) (Supplementary Table S2) [19].

The incidence of serious complications due to PCI during ischemia-driven rehospitalization was 2 of 15 (13.4%) in the CR group and 8 of 45 (17.7%) in the IR group. Complete percutaneous revascularization with PCI was achieved in 17 (37.8%) patients in the IR group who returned for rehospitalization. The remaining patients in the IR group were advised to undergo CABG or optimal medical therapy. The incidence of all serious complications (CR and IR together) due to PCI during ischemia-driven rehospitalization was statistically significantly higher: 10 (16.7%), compared to primary hospitalization, 19 (8.1%) (OR 2.83; 95% CI 1.091–6.972; *p* = 0.019) (Supplementary Table S3).

## 4. Discussion

Our all-comer long-term follow-up study, which included patients with STEMI and MVD, revealed the following main findings: (1.) The primary composite endpoint of ischemia-driven rehospitalization and CV mortality was higher in the IR than in the CR group during follow-up time (47.3% vs. 32.9%, log-rank *p* = 0.025), which was driven by CV mortality (23.6% vs. 12.9%, log-rank *p* = 0.047) since there was no difference in ischemia-driven rehospitalizations (log-rank *p* = 0.206). (2.) Rehospitalization did not influence CV mortality in the CR group (*p* = 0.49), while it significantly impacted CV mortality in the IR group (*p* = 0.03) with better CV survival of IR patients who came back for rehospitalization. (3.) After adjustments for confounders, the revascularization method did not significantly influence the primary endpoint, but the primary combined endpoint occurrence increased with age (*p* = 0.014), diabetes (*p* = 0.006), chronic kidney disease (*p* = 0.001), cardiogenic shock at presentation (*p* = 0.003), CTO (*p* = 0.046), and the need for ischemia-driven rehospitalization (*p* < 0.0001). (4.) There was no significant increase in the risk of the primary combined endpoint with IR (HR 1.25, *p* = 0.385). Nevertheless, the following factors significantly increased the risk of the primary combined endpoint: a residual SYNTAX I score greater than 5.5 (HR 1.73, *p* = 0.017), age over 70 (HR 1.94, *p* = 0.004), being female (HR 1.88, *p* = 0.008), having stage 4 or 5 chronic kidney disease (HR 2.97, *p* = 0.001), and cardiogenic shock at the time of inclusion (HR 3.16, *p* < 0.001). Consequently, it is essential to employ a meticulous patient-tailored approach when managing STEMI patients with MVD, and, if they undergo IR revascularization, a thorough patient follow-up should be planned.

The most effective treatment for STEMI patients is PCI; however, the data regarding the timing of treatment for non-culprit lesions is inconsistent due to the fact that up to 50% of STEMI patients have significant MVD. The registry data on this matter vary from the harm of performing CR during primary PCI or index hospitalization to the benefit in particular subgroups and overall [2,3,4,5,21,22]. Since registries usually use less restrictive inclusion and exclusion criteria and set harder endpoints as all-cause or CV mortality, they may represent an important aspect of research in this field. Our all-comer study showed lower all-cause and CV mortality of CR patients during index hospitalization, and IR was an independent risk factor for all-cause mortality but not for CV mortality [19]. After adjustment for confounders, the benefit of CR disappeared, with the strongest independent predictors of mortality being age, diabetes, higher than normal serum creatinine concentration, cardiogenic shock at presentation, and CTO [19].

In the last decade, several randomized trials were conducted to address the revascularization question of STEMI and MVD patients [6,7,8,9,10]. Nevertheless, it is important to notice that those trials did not include patients with severe left main coronary artery stenosis, advanced age, limited life expectancy, cardiogenic shock, renal insufficiency, previous MI, PCI, or CABG, or patients with CTOs. Most of the prospective randomized trials performed were positive on their combined outcome—usually a combination of all-cause or CV mortality and major adverse events and the need for revascularization—and showed the better outcome of complete revascularization either during primary PCI or postponed procedure during the same hospitalization guided angiographically or with FFR [6,7,8,9]. One of the main criticisms of those trials was that their positive results were primarily due to refractory angina, which required additional rehospitalization and revascularization of non-culprit lesions as part of their primary endpoint. It is intriguing that one randomized trial was entirely negative and did not even demonstrate a trend toward improved outcomes in CR patients, as their combined outcome consisted of all-cause mortality, nonfatal MI, and stroke. Additionally, they designated a patient as CR if they underwent revascularization of non-culprits within 40 days of the culprit PCI [23]. Bravo et al. conducted one of the largest meta-analyses, concluding that CR may be superior. However, their confidence in the effect estimate was limited due to the low quality of evidence and the ongoing need for further research to confirm or refute the potential difference between CR and IR [24]. Recently, the results from the heavily expected, largest randomized trial to date (COMPLETE trial) on the topic were published and showed CR reduced the combined primary outcome (CV death or myocardial infarction) by 26% (*p* = 0.004, number needed to treat (NNT) = 37) and the secondary combined outcome (CV death, myocardial infarction, revascularization) by 46% (*p* < 0.001, NNT = 13) [10]. It is interesting that our study showed a lower occurrence of the combined endpoint of ischemia-driven rehospitalization and CV mortality, but after adjusting for confounders, the primarily composed endpoint occurrence was independent of the revascularization method, and the real determinants were age (*p* = 0.014), diabetes (*p* = 0.006), chronic kidney disease (*p* = 0.001), cardiogenic shock at presentation (*p* = 0.003), chronic total occlusion (*p* = 0.046), and the need for ischemia-driven rehospitalization (*p* < 0.0001). Additionally, IR did not significantly alter the risk of occurrence of the combined primary endpoint (HR 1.25, *p* = 0.385); however, having a residual SYNTAX I score higher than 5.5 (HR 1.73, *p* = 0.017), being female (HR 1.88, *p* = 0.008), being older than 70 (HR 1.94, *p* = 0.004), having stage 4 or 5 chronic kidney disease (HR 2.97, *p* = 0.001), and having cardiogenic shock at inclusion (HR 3.16, *p* < 0.001) significantly increased the risk of these outcomes. While this is consistent with a previously published study that assessed the residual SYNTAX I score for target vessel failure following the implantation of a drug-eluting stent, the results cannot be directly compared because the study excluded patients with an acute myocardial infarction [25]. Different studies propose different residual SYNTAX I score cutoff values for different endpoints, but we agree that the higher the residual SYNTAX I score, the greater the risk of future events [26,27]. For CR patients, a residual SYNTAX I score < 6 was predictive of better primary and secondary outcomes in the COMPLETE study, but a residual SYNTAX I score ≥ 6 was predictive solely of a secondary outcome [10].

Since we have growing evidence that CR may be better than IR for STEMI and MVD patients, we wanted to see if that also shows on ischemia-driven rehospitalizations. Rehospitalizations of patients after STEMI are not so rare and range from 10 to 20% in the first 30 days after release from the hospital [28,29,30]. Not all readmissions are due to ischemia or heart failure but also due to procedural complications, comorbidities, and the patient’s or physician’s fear of MI recurrence [28,29,30]. Patients with severe non-culprit lesions are frequently readmitted through the emergency department or outpatient clinic when they are experiencing ischemic symptoms [28,29,30]. Recent meta-analyses showed that immediate complete revascularization reduces recurrent MI and repeat revascularization compared to IR and staged revascularization, while there is no impact on CV mortality [31]. We expected that patients in the IR group would have had more readmissions than the CR group due to possible ischemia, but surprisingly the ischemia-driven rehospitalization rate was not different during the whole follow-up time (*p* = 0.206), and the reason for readmission did not differ between the CR and IR groups (*p* = 0.519).

Readmissions after STEMI pose a great financial burden to healthcare systems, and there is growing evidence that CR may improve survival, MI recurrence, and the need for repeat revascularization [28,29]. Nevertheless, STEMI patients with MVD are a diverse population with a variety of comorbidities and risk factors. Various scoring systems have been employed by researchers to identify patients who could potentially benefit the most from CR in this context. The GRACE score is the most well-known, and there are also others, particularly for STEMI patients and MVD [30]. In our study, ischemia-driven rehospitalization did not affect the CV survival of CR patients (log rank *p* = 0.49), while it significantly impacted the survival of IR patients (log rank *p* = 0.03). More than one rehospitalization did not influence the CV survival of IR patients (log-rank *p* = 0.83).

Since revascularization with PCI is an invasive procedure, we also analyzed the complication rate due to PCI during rehospitalization in comparison to revascularization at index hospitalization. The complication rate due to percutaneous revascularization was significantly higher than at index hospitalization (*p* = 0.019), which may reflect why some patients in the IR group were not completely revascularized during index hospitalization.

The main strengths of this study are the long follow-up, inclusion of patients treated in routine clinical practice, and choice of hard endpoints such as ischemia-driven rehospitalization and CV mortality. Nevertheless, it is imperative to acknowledge the study’s constraints. The inclusion of a relatively small number of patients in this single-center retrospective study may be susceptible to selection bias, missing data, and underreporting of events. It is impossible to eliminate the impact of residual confounding. In the IR group, ischemia was not systematically assessed, and no data were collected from patients who did not present for rehospitalization during the follow-up period. If myocardial ischemia was systematically evaluated, the IR group may have experienced a greater number of planned ischemia-driven rehospitalizations. The ischemia of non-culprit lesions was not regularly assessed, and most PCI was guided only by the severity of coronary lesions and clinical presentation.

## 5. Conclusions

Patients with STEMI and MVD in our retrospective study treated with CR had lower rates of the combined endpoint of ischemia-driven rehospitalization and CV mortality, but after adjusting for confounders, the occurrence of the primary composed endpoint was independent of the revascularization method. On its own, IR was not a substantial risk factor for ischemia-driven rehospitalization and CV mortality. However, residual SYNTAX I score, older age, female gender, chronic kidney disease, and cardiogenic shock were. Ischemia-driven rehospitalization significantly impacted CV mortality of the IR group, with better survival of those who came for rehospitalization. According to our study, patients with STEMI and MVD with incomplete revascularization should be carefully followed up since they have a worse prognosis in the long term.

## Figures and Tables

**Table 1:** Patient and procedural characteristics.

Variable	CR *N* = 70	IR *N* = 165	*p*-Value
Age ≥ 61 years, *n* (%)	41 (59)	114 (69)	0.160
Men, n (%)	49 (70)	120 (73)	0.790
Arterial hypertension, n (%)	43 (61)	116 (70)	0.239
Diabetes, n (%)	11 (16)	42 (25)	0.143
Current smoker, n (%)	26 (37)	57 (35)	0.817
Hyperlipidemia, n (%)	46 (66)	104 (63)	0.808
Family history of cardiovascular disease *, n (%)	11 (16)	30 (18)	0.789
Chronic kidney disease, n (%)	6 (9)	23 (14)	0.354
Previous myocardial infarction, n (%)	4 (6)	23 (14)	0.077
Previous PCI, n (%)	5 (7)	13 (8)	1.000
Previous CABG, n (%)	0 (0)	6 (4)	0.183
Coronary intervention			
Culprit artery			
Left descending coronary	26 (37)	57 (35)	0.766
Right coronary	36 (51)	81 (49)	0.424
Left circumflex coronary	8 (11)	27 (16)	0.777
Number of significant stenoses of non-culprit artery			0.005
1	38 (54)	56 (34)	
>1	32 (46)	109 (66)	
CTO	1 (1)	29 (18)	<0.001
After CABG		4 (2)	0.321
Number of PCI procedures †			
1	32 (46)	130 (79)	<0.001
>1	38 (54)	35 (21)	<0.001
Integrilin use	13 (19)	34 (21)	0.859
Intra-aortic balloon pump	5 (7)	26 (16)	0.092
Transfusion due to coronary intervention complication	4 (6)	8 (5)	0.754
LVEF after PCI †			0.871
˃55%	20 (29)	40 (24)	
45 to ≤54%	7 (10)	21 (13)	
30 to ˂45%	7 (10)	13 (8)	
˂30%	3 (4)	10 (6)	

Legend: * <55 years in men and <65 years in women; † during index hospitalization. CABG: coronary artery bypass graft; CR: complete revascularization; CTO: chronic total occlusion; IR: incomplete revascularization; LVEF: left ventricular ejection fraction; PCI: percutaneous coronary intervention; TIMI: Thrombolysis In Myocardial Infarction flow.

**Table 2:** The method of myocardial revascularization and the cause of ischemia-driven rehospitalization in the CR and IR groups.

	CR (N = 70)	IR (N = 165)	Fisher Test
Reason for Rehospitalization	*N* (%)	*N* (%)	*p*-Value
AP	6 (8.6)	11 (6.7)	0.519
NAP	3 (4.3)	5 (3.0)	
STEMI	1 (1.4)	8 (4.8)	
NSTEMI	1 (1.4)	5 (3.0)	
Heart failure	1 (1.4)	6 (3.6)	
Other	3 (4.3)	10 (6.1)	
With rehospitalization	15 (21.4)	45 (27.3)	
Without rehospitalization	55 (78.6)	120 (72.7)	
**Method of revascularization**			
CABG	1 (1.4)	5 (3.0)	0.672
PCI LM	0 (0.0)	1 (0.6)	1.000
PCI LAD	4 (5.7)	11 (6.7)	1.000
PCI LCX	1 (1.4)	10 (6.1)	0.181
PCI RCA	2 (2.9)	8 (4.8)	0.727
Adjustment of treatment with medications	7 (10.0)	10 (6.1)	0.257

Legend: AP: angina pectoris; CR: complete percutaneous revascularization; CABG: coronary artery bypass grafting; IR: incomplete percutaneous revascularization; LAD: left anterior descending coronary artery; LCX: left circumflex coronary artery; LM: left main coronary artery; NAP: unstable angina pectoris; NSTEMI: Non-ST elevation myocardial infarction; PCI: percutaneous coronary intervention with dilation of the coronary artery; RCA: right coronary artery; STEMI: ST-elevation myocardial infarction.

**Table 3:** Independent predictors of ischemia-driven rehospitalization and cardiovascular mortality in the CR vs. IR group (Cox multivariate model *).

Comparators and Risk Factors	Ischemia-Driven Rehospitalization and CV Mortality		
	Coefficient	HR (95% CI)	* p * -Value
IR	−0.19	0.83 (0.39–1.80)	0.622
CR	0.19	1.21 (0.56–2.54)	0.622
Age	0.04	1.04 (1.00–1.08)	0.014
Smoker	−0.26	0.77 (0.35–1.67)	0.507
Diabetes	1.13	3.08 (1.36–6.78)	0.006
LDL	−0.03	0.97 (0.66–1.39)	0.860
Creatinine value at inclusion (natural logarithm)	0.01	1.01 (1.00–1.01)	0.001
Previous MI	−0.08	0.92 (0.21–3.31)	0.909
Previous PCI	0.43	1.54 (0.27–8.26)	0.612
Previous CABG	1.16	3.18 (0.15–22.58)	0.319
Cardiogenic shock	1.38	3.97 (1.57–9.66)	0.003
CTO	0.99	2.70 (0.96–6.94)	0.046
Residual SYNTAX I score	0.01	1.01 (0.95–1.07)	0.692
Rehospitalization †	2.08	8.03 (3.85–17.52)	<0.0001

* Adjusted by the following confounders: age, smoker, diabetes, LDL, chronic kidney disease, previous myocardial infarction or percutaneous coronary revascularization, CABG before inclusion, cardiogenic shock at inclusion or the need for intra-aortic balloon pump ≤15 days after inclusion, CTO, residual SYNTAX I score, and ischemia-driven rehospitalization; † ischemia-driven rehospitalization. Legend: CI: confidence interval; CR: complete percutaneous revascularization; CABG: coronary artery bypass graft operation; HR: hazard ratio; IR: incomplete percutaneous revascularization; LDL: low-density lipoprotein serum concentration; MI: myocardial infarction; PCI: percutaneous coronary intervention; SYNTAX I: Synergy between PCI with Taxus and Cardiac Surgery Score I.

## Data Availability

Data may be acquired upon contacting the corresponding author.

## Supplementary Materials

The following supporting information can be downloaded at: https://www.mdpi.com/article/10.3390/jcm14134793/s1.
