# Supplementary material for: Effect of Complete Revascularization in STEMI: Ischemia-Driven Rehospitalization and Cardiovascular Mortality
Source: J Clin Med. 2025 Jul 7;14(13):4793. doi: 10.3390/jcm14134793 (PMC12250939; doi:10.3390/jcm14134793)
Supplement: Supplementary file 1 [file jcm-14-04793-s001.zip › jcm-3595090-supplementary.pdf]

Supplementary table S1. Cardiovascular therapies and laboratory variables.

Variable	CR<br>*N* = 70	IR<br>*N* = 165	*p*-value
**Therapy before admission, *n* (%)**			
Antiplatelet (aspirin or clopidogrel)	0 (0%)	4 (2.4%)	0.321
Anticoagulant (warfarin or non-vitamin K antagonist oral anticoagulant)	9 (12.9%)	27 (16.4%)	0.558
Dual antiplatelet	1 (1.4%)	1 (0.6%)	0.508
Beta-blocker	12 (17.1%)	33 (20%)	0.718
Calcium channel blocker	8 (11.4%)	27 (16.4%)	0.424
Angiotensin-converting enzyme inhibitor	16 (22.9%)	55 (33.3%)	0.122
Angiotensin receptor blocker	7 (10.0%)	19 (11.5%)	0.823
Mineralocorticoid receptor blocker	0 (0%)	3 (1.8%)	0.556
Statin	13 (18.6%)	39 (23.6%)	0.492
Ezetimibe	1 (1.4%)	1 (0.6%)	0.508
**Therapy at discharge/transfer to regional hospital, *n* (%)**			
Aspirin	70 (100%)	165 (100%)	1.000
Clopidogrel	67 (95.7%)	149 (90.3%)	0.199
Prasugrel	3 (4.3%)	7 (4.2%)	1.000
Beta-blocker	47 (67.1%)	85 (51.5%)	0.031
Calcium-channel blocker	4 (5.7%)	8 (4.8%)	0.754
Angiotensin-converting enzyme inhibitor	57 (81.4%)	113 (58.6%)	0.055
Angiotensin receptor blocker	5 (7.1%)	4 (2.4%)	0.131
Mineralocorticoid receptor blocker	9 (12.9%)	16 (9.7%)	0.492
Statin	68 (97.1%)	138 (83.6%)	0.004
Ezetimibe	3 (4.3%)	3 (1.8%)	0.366
**Laboratory variables, mean ± SD**			
Maximum troponin I ultra value during index hospitalization (μg/L)	(n=60)<br>72.7±80	(n=150)<br>99±115.6	0.385
Low-density lipoprotein cholesterol (mmol/L)	(n=40)<br>3.3±1.3	(n=86)<br>3.2±1.2	0.975
Blood hemoglobin (g/L)			
Before or at index intervention	(n=56)<br>130.1±18.5	(n=129)<br>129.9±17.4	0.702
Lowest concentration during index hospitalization	(n=44)<br>126.5±19.3	(n=99)<br>127.1±15.8	0.894
Blood creatinine (μmol/L)			
Before or at index intervention	(n=56)<br>84.0±26.0	(n=135)<br>112.5±95	0.078
Highest concentration after PCI during index hospitalization	(n=57)<br>87.6±51.1	(n=123)<br>105±87.8	0.487

Legend: CR: complete revascularization; IR: incomplete revascularization; PCI: percutaneous coronary intervention.

Supplementary table S2. Complications during index hospitalization.

Variable	CR<br>(*N* = 70)	IR<br>(*N* = 165)	p-value

Complications, *n* (%)	3 (4.3)	16 (9.7)	0.199
Malignant arrhythmias	1 (1.4)	4 (2.4)	
Acute stent thrombosis	2 (2.9)	1 (0.6)	
Coronary artery perforation	0 (0)	3 (1.8)	
TIMI 0 or 1 flow	0 (0)	3 (1.8)	
Coronary artery dissection	0 (0)	2 (1.2)	
Complications due to puncture site	0 (0)	3 (1.8)	
Mechanical complications	0 (0)	1 (0.6)	

Legend: CR: complete percutaneous revascularization; IR: incomplete percutaneous revascularization; TIMI: Thrombolysis and Myocardial Infarction.

Supplementary table S3. Complications during ischemia-driven rehospitalization.

**Variable**	**CR (*N* = 15) (%)**	**IR (*N* = 45) (%)**	**CR during rehosp. (*N* = 17) (%)**
Complications, *n* (%)	2 (13.4)	8 (17.7)	3 (17.6)
Malignant arrhythmias	0 (0)	0 (0)	0 (0)
Acute stent thrombosis	0 (0)	1 (2.2)	0 (0)
Coronary artery perforation	0 (0)	0 (0)	0 (0)
TIMI 0 to 1 flow	0 (0)	0 (0)	0 (0)
Coronary artery dissection	1 (6.7)	2 (4.4)	0 (0)
Complications due to puncture site	0 (0)	0 (0)	0 (0)
Periprocedural MI	0 (0)	1 (2.2)	1 (5.9)
Other	0 (0)	4 (8.9)	2 (11.8)
Mechanical complications	1 (6.7)	0 (0)	0 (0)

Legend: CR: complete percutaneous revascularization; IR: incomplete percutaneous revascularization; CR during rehosp.: Subjects in the IR group who underwent complete percutaneous revascularization on rehospitalization; TIMI: Thrombolysis In Myocardial Infarction.

Supplementary table S4: The severity of non-culprit stenosis, residual SYNTAX I score, and coronary stent characteristics.

	**CR group (N=70)**			**IR group (N=165)**			***p*-value**
**Variable**	***N***	**Mean ± SD**	**Median (Q1, Q3)**	***N***	**Mean ± SD**	**Median (Q1, Q3)**	
Residual SYNTAX I score	70	6.4 ± 4.5	5 (2.3, 7.8)	165	8.7 ± 6.4	7.0 (4.0, 13.0)	0.011
Non-culprit stenosis severity (%)	70	83.6 ± 12.3	85.0 (80.0, 95.0)	165	79.7 ± 13.4	78.3 (70.0, 90.0)	0.015
Number of stents implanted per patient							
Drug eluting	32	1.8 ± 1.0	2 (1.0, 2.0)	37	2.0 ± 1.1	2 (1.0, 2.0)	0.667

Bare metal	62	2.4 ± 1.4	2 (1.0, 3.0)	129	2.1 ± 1.3	2 (1.0, 3.0)	0.057

Legend: CR: complete revascularization; IR: incomplete revascularization; Q: quartile; SD: standard deviation; SYNTAX I: Synergy Between PCI With Taxus and Cardiac Surgery score I.

Supplementary figure S1: Estimated survival by residual SYNTAX I score group

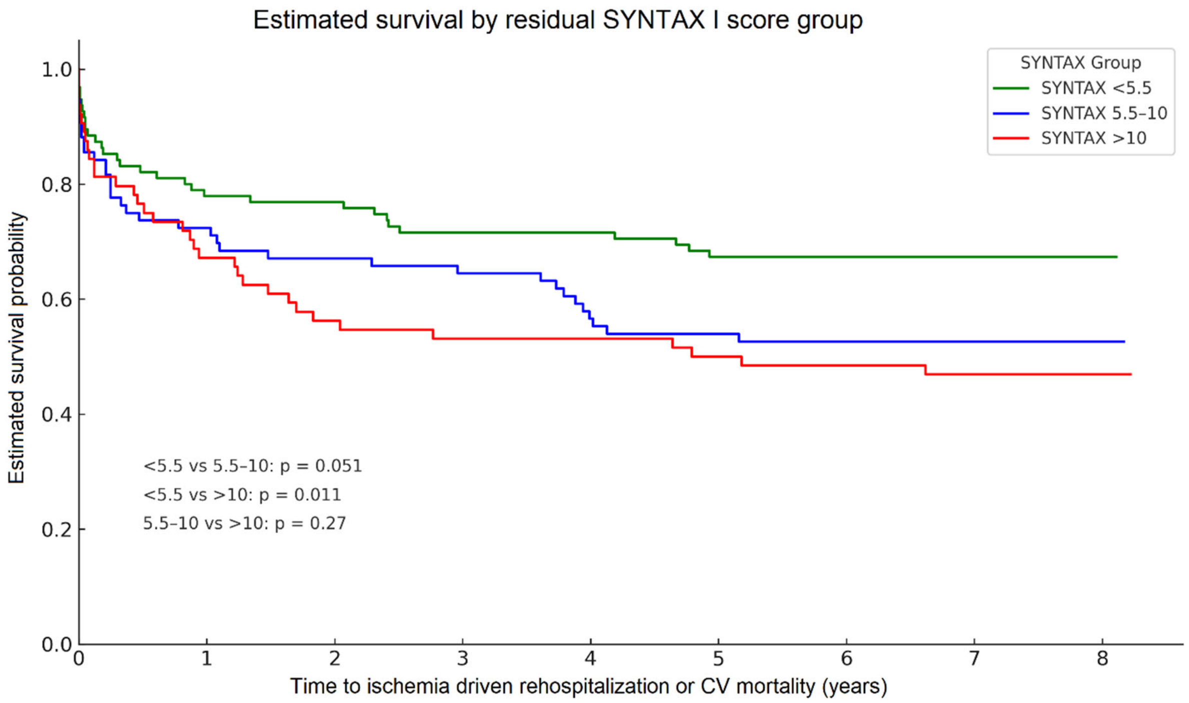

Supplementary table S5: Risk analysis by residual SYNTAX I score groups for the composite endpoint of ischemia-driven rehospitalization or CV mortality using a logistic regression approximation.

**Residual SYNTAX I Score Group**	**Odds Ratio (OR)**	**95% CI**	**p-value**
< 5.5 (reference)	–	–	–
5.5–10	1.86	0.998 - 3.46	0.051
> 10	2.34	1.22 - 4.49	0.011

Legend: CI: Confidence interval; SYNTAX I: Synergy Between PCI With Taxus and Cardiac Surgery score I.
